# Epimorphin Alters the Inhibitory Effects of SOX9 on Mmp13 in Activated Hepatic Stellate Cells

**DOI:** 10.1371/journal.pone.0100091

**Published:** 2014-06-27

**Authors:** James Pritchett, Varinder S. Athwal, Emma Harvey, Katherine Martin, Jessica Llewellyn, Philip Ireland, Alexander Nicolaides, Martin J. Humphries, Nicoletta Bobola, Neil A. Hanley, Karen Piper Hanley

**Affiliations:** 1 Centre for Endocrinology and Diabetes, Manchester Academic Health Sciences Centre, University of Manchester, Manchester, United Kingdom; 2 School of Dentistry, Manchester Academic Health Sciences Centre, University of Manchester, Manchester, United Kingdom; 3 Manchester Biomedical Research Centre, Central Manchester University Hospitals NHS Foundation Trust, Manchester, United Kingdom; 4 Wellcome Trust Centre for Cell-Matrix Research, University of Manchester, Manchester, United Kingdom; Institute of Hepatology, Foundation for Liver Research, United Kingdom

## Abstract

**Background and Aims:**

Liver fibrosis is a major cause of morbidity and mortality. It is characterised by excessive extracellular matrix (ECM) deposition from activated hepatic stellate cells (HSCs). Although potentially reversible, treatment remains limited. Understanding how ECM influences the pathogenesis of the disease may provide insight into novel therapeutic targets for the disease. The extracellular protein Epimorphin (EPIM) has been implicated in tissue repair mechanisms in several tissues, partially, through its ability to manipulate proteases. In this study, we have identified that EPIM modulates the ECM environment produced by activated hepatic stellate cells (HSCs), in part, through down-regulation of pro-fibrotic Sex-determining region Y-box 9 (SOX9).

**Methods:**

Influence of EPIM on ECM was investigated in cultured primary rat HSCs. Activated HSCs were treated with recombinant EPIM or SOX9 siRNA. Core fibrotic factors were evaluated by immunoblotting, qPCR and chromatin immunoprecipitation (ChIP).

**Results:**

During HSC activation EPIM became significantly decreased in contrast to pro-fibrotic markers SOX9, Collagen type 1 (COL1), and α- Smooth muscle actin (α-SMA). Treatment of activated HSCs with recombinant EPIM caused a reduction in α-SMA, SOX9, COL1 and Osteopontin (OPN), while increasing expression of the collagenase matrix metalloproteinase 13 (MMP13). Sox9 abrogation in activated HSCs increased EPIM and MMP13 expression.

**Conclusion:**

These data provide evidence for EPIM and SOX9 functioning by mutual negative feedback to regulate attributes of the quiescent or activated state of HSCs. Further understanding of EPIM's role may lead to opportunities to modulate SOX9 as a therapeutic avenue for liver fibrosis.

## Introduction

Fibrosis of the liver is a major cause of worldwide morbidity and mortality. End-stage disease can be treated by transplantation: however, there is a huge shortage of donor organs and focus on end-stage disease is too late. Urgent development of novel antifibrotic therapies is needed during reversible phases of the disease but none have been so far approved [1], [2].

Liver fibrosis is a result of repetitive rounds of injury and attempted repair resulting in excessive extracellular matrix (ECM) deposition. One of the key cells mediating fibrosis is the hepatic stellate cell (HSC) [3], [4]. Following injury to the liver, HSCs become activated into proliferative myofibroblasts (MFs), secrete damaging ECM and infiltrate the surrounding tissue to cause fibrosis [3], [4]. This process is regulated by multiple pathways and factors, including the transcription factor SOX9 (sex determining region Y-box 9) [5]. In response to pro-fibrotic cytokines, SOX9 becomes expressed in activated HSCs [6] where it regulates production of Collagen type 1 (COL1), the major collagen associated with the fibrotic scar [6], and Osteopontin (OPN) [7], a potential biomarker of liver disease and implicated in the progression of fibrosis [7]–[11]. In addition, factors capable of degrading matrix, matrix metalloproteinases (MMPs), and their inhibitors (tissue inhibitors of matrix metalloproteinases; TIMPs) are also altered to favor ECM accumulation [12]. Given its central importance, understanding how ECM components influence the pathogenesis of fibrosis may provide insights into potential therapeutic strategies for liver fibrosis.

An ECM protein which has received attention as a modulator of tissue repair is Epimorphin (EPIM). Originally identified as a mesenchymal cell-surface associated protein [13], EPIM (also known as syntaxin 2) plays significant morphogenic roles in several tissues including skin, hair follicle, mammary gland, lung, kidney, intestine, pancreas and liver [14]–[16]. Although complex, evidence suggests EPIM has two distinct functions depending on its location. As an intracellular protein it is involved in vesicular transport. As an extracellular protein EPIM acts as a morphogen capable of signaling through the epidermal growth factor receptor (EGFR) and integrins [17].

In the adult liver, EPIM is expressed in the connective tissue surrounding blood vessels, along the sinusoidal lining where HSCs reside [18], [19] and in mesenchyme surrounding the bile duct where it is thought to play a role in duct formation [15], [20]. In vivo, there is a reduction in EPIM expression following liver injury and HSC activation [21]. However, in models of liver regeneration, Epim also appears to increase later in HSCs highlighting a potential role during the recovery phase of tissue repair [21]–[23]. In line with this, a feature of EPIM in both renal and liver fibrosis is its ability to induce protease expression favoring collagen degradation and resolution of fibrosis [22], [24], [25]. Although this provides clues of EPIM's role in modulating the ECM in fibrosis the mechanisms by which it acts and is regulated are less clear.

In this study, we hypothesized that the role of EPIM to ameliorate fibrosis is mediated at least in part by an interplay with SOX9. We have investigated this in vitro in HSCs to demonstrate an inter-dependence governed by negative feedback that could be the focus of future work aimed a therapeutic intervention in liver fibrosis.

## Methods

### Primary cell culture

Primary rat hepatic stellate cells (rHSCs) were isolated and cultured as described previously [6], [26] under project licence 40/3417 following approval by the University of Manchester local Ethical Review Committee. *Sox9* gene silencing was carried out using siRNA by Nucleofection (Amaxa Biosystems GmbH) as described previously [6]. For time course activation, cells were either immediately taken for protein analysis (time 0) or maintained in culture without passage for the desired time (3, 7 and 10 days) [6], [7], whereas all other experiments (Chromatin immunoprecipitation, siRNA BrdU and EPIM) used culture-activated (7 days on tissue culture plastic, in 16% serum) rHSCs, passaged once. Following passage, rHSCs were treated for 24 hours with recombinant human EPIM (R&D Systems) or vehicle control (PBS) in 0% serum at 0.5 µg/ml, 1 µg/ml or 2 µg/ml or cultured on Matrigel substrate (6.6 mg/ml) for 2 weeks [27]. For BrdU incorporation, HSCs (activated and passaged as above) were plated onto fibronectin coated (5 µg/cm^2^; Sigma-Aldrich) glass chamber slides and left overnight in 16% serum [6]. HSCs were then cultured in serum free media in the presence or absence of 2 µg/ml rhEPIM (as above) with BrdU (30 µM) incorporation for the final 4 hours (in a total 24 hour EPIM treatment experiment). HSCs were washed and fixed in 4% PFA for immunohistochemistry as previously described [6]. BrdU positive cells were counted from 5 separate areas of each culture chamber at 10× magnification and numbers expressed as a percent of total cells counted per area.

### Animal Models of Liver Fibrosis

All *in vivo* experiments were carried out under UK Home Office Licence 40/3417 and approved by the University of Manchester local ethical review process. Male C57Bl/6J mice were given twice weekly intraperitoneal injections of carbon tetrachloride (CCl_4_) diluted 1∶3 in olive oil, or vehicle control (2 µl/mg body weight) for 8 weeks. At the appropriate time point animals were killed by CO_2_ asphyxiation and liver and serum samples were prepared for further analysis. Fibrosis was assessed from serum liver enzymes activities and histological Picrosirius Red (PSR) for total collagen and α-SMA staining for myofibroblasts, as previously described [28].

### Gene and Protein expression

For quantitative PCR (qPCR), RNA was isolated from cells using the RNeasy kit (Qiagen Ltd., West Sussex, UK). Following DNase I treatment, cDNA was synthesized from 1 µg of RNA with a RNA-to-cDNA kit (Applied Biosystems Ltd., Cheshire, UK). qPCR reactions were carried out on a StepOnePlus Real-Time PCR system (Applied Biosystems Ltd) using 1 µl of cDNA, intron-spanning primers wherever possible (Table S1), and SYBR green (PrimerDesign Ltd., Southampton, UK). Changes in mRNA expression were calculated using ΔΔCT methodology relative to both GusB and β-actin [7], [29].

Immunoblotting and quantification were performed as described previously [6], [7], using denaturing SDS-PAGE, followed by transfer to nitrocellulose. Antibodies were diluted in PBS containing 0.1% tween and 5% milk powder. Membranes were probed with the appropriate HRP conjugated secondary antibody (GE Healthcare) and proteins visualised using chemiluminescence (ECL Prime, GE Healthcare). Qauntification by densitometry was carried out using Qunatity One software (Biorad). Antibodies used are listed in Table S2.

### Chromatin immunoprecipitation

Chromatin immunoprecipitation (ChIP) assays were performed as described previously [7]. Briefly, sheared chromatin was isolated from rHSCs using an anti-SOX9 antibody (Santa Cruz Biotechnology Inc, CA). PCR was then performed using primers to amplify the conserved upstream element of *Mmp13* (Forward: TCCACAGCAAACACAAG Reverse: TTCATTCAAATATAAGAGTCG). Fold enrichment of the conserved region in SOX9 ChIP samples versus negative control IgG ChIP was calculated relative to input chromatin, using ^ΔΔ^Ct following qPCR as described elsewhere [30]. To quantify the effect of rhEPIM treatment on Sox9 binding to the conserved region of *Mmp13*, fold change in enrichment (% input) was calculated following qPCR in control versus rhEPIM treated ChIP samples.

### Statistical analysis

All experiments were carried out three times or more and, in the case of rHSCs, were investigated in different preparations of stellate cells from different animals. Data are expressed as means ± SE and statistical significance was determined using two-tailed Student's *t* test where p<0.05 was considered significant.

## Results

### Epimorphin expression is decreased during activation of HSCs

**Figure 1:**
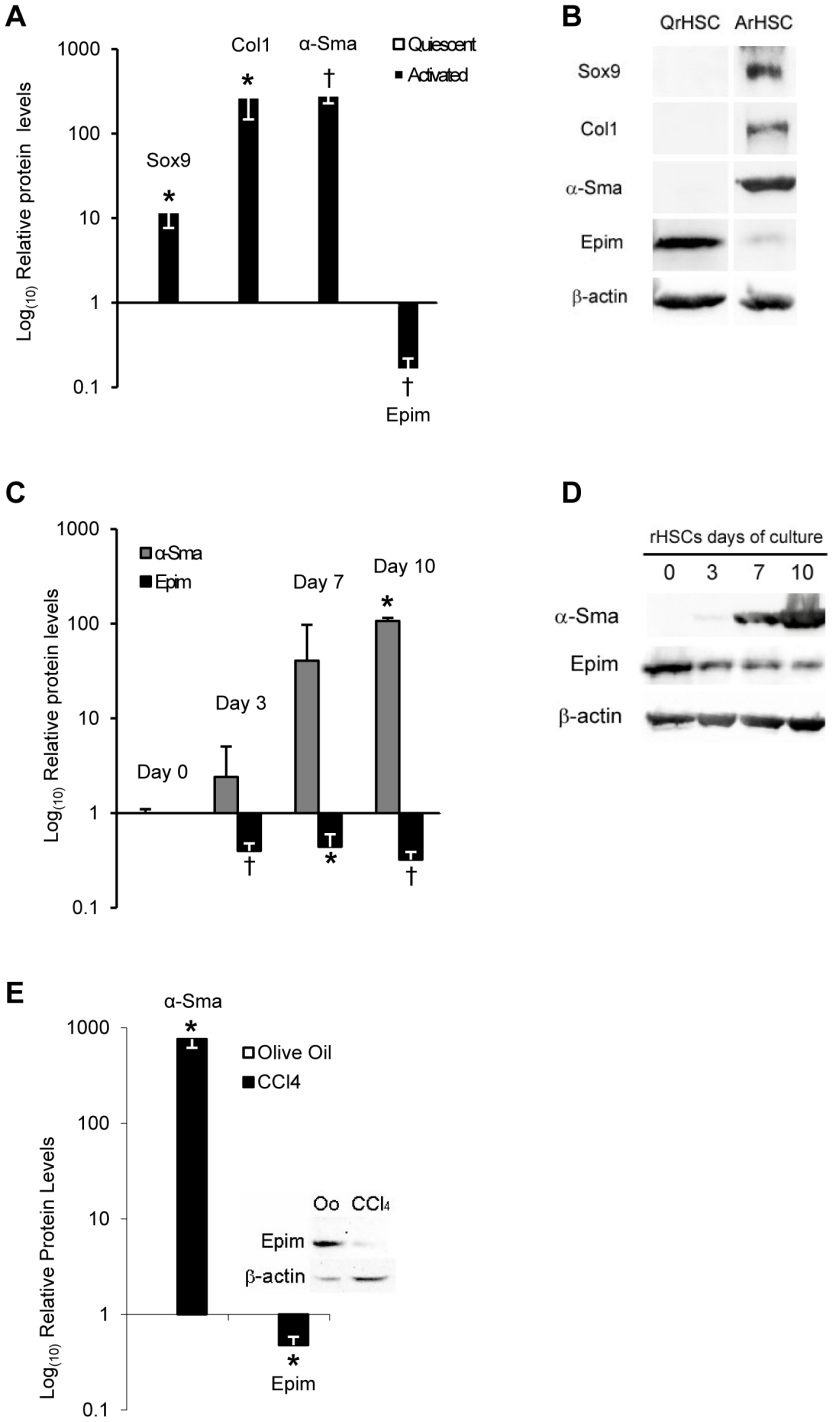
EPIM expression in activated HSCs and fibrotic liver. Quantification of proteins in rat HSCs and mouse whole liver lysates (rHSCs; A and C, mouse liver; E) and immunoblotting (rHSCs; B and D, mouse liver; E). (A) Induction of SOX9, COL1 and α-SMA with a reduction in EPIM in activated rat HSCs. Example immunoblot shown in (B). (C) Time course activation of rHSCs showing reduced EPIM levels in contrast to α-SMA (relative to day 0 quiescent; set at 1). Example immunoblot shown in (D). (E) Increased α-SMA protein in liver lysate from CCl_4_ treated mice correlated with reduced EPIM protein levels (olive oil control is set at 1). Example EPIM immunoblot shown (inset E). α-SMA immunoblot shown in Figure 1B. All quantification was normalized to β-actin. *, p<0.05, †, p<0.005.

We initially investigated the expression of EPIM in quiescent and 7 day activated rHSCs (Fig. 1A & B). In contrast to increased production of SOX9, α- Smooth muscle actin (α-SMA) and COLI proteins, we detected an 84% decrease in full length 34 KDa EPIM in activated rHSCs (Fig. 1A & B). The EPIM levels of quiescent rHSCs were reduced by 3 days of activation, before cells were fully activated as shown by intermediate levels of α-SMA (Fig. 1C & D) SOX9, COL1 and OPN [6], [7]. Moreover, compared to control, EPIM was almost absent in protein lysates from mice with fibrosis induced by CCl_4_ injection (Fig. 1E; Figure S1).

### Epimorphin decreases expression of pro-fibrotic genes in HSCs

**Figure 2:**
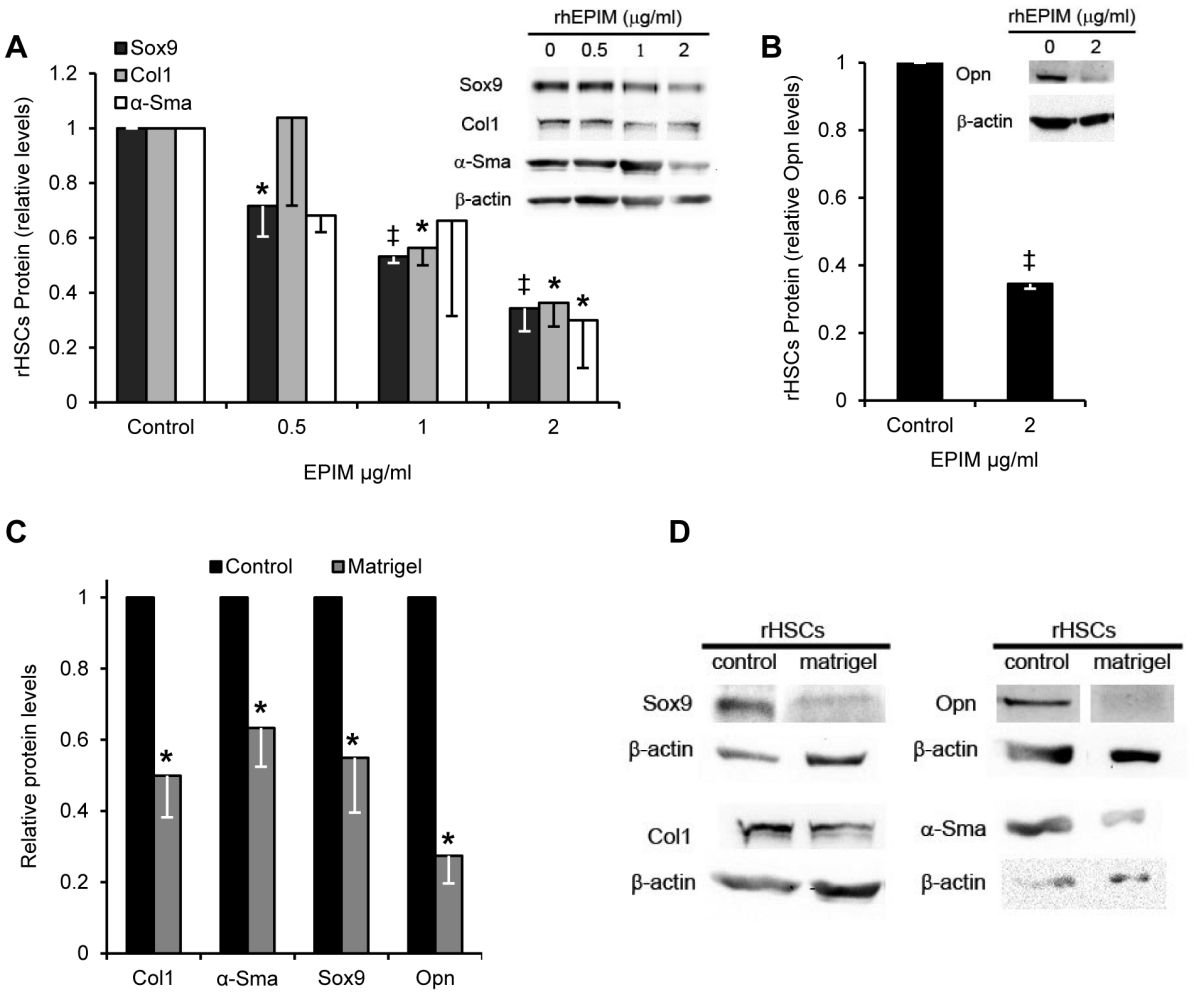
EPIM alters the pro-fibrotic profile of activated HSCs. (A and B) Quantification of pro-fibrotic proteins showing decreased SOX9, COL1 and α-SMA in activated rHSCs following rhEPIM treatment. (B) Highest rhEPIM dose reduced OPN protein by ∼65%. Example immunoblot for quantification shown (inset in A and B). (C and D) Quantification (C) and immunoblot (D) indicating decreased expression of COL1, α-SMA, SOX9 and OPN following rHSCs culture on Matrigel for 14 days. All immunoblotting quantification was normalized to β-actin. *, p<0.05, ‡, p<0.001.

EPIM's association with the quiescent HSC phenotype [18], [21] and its role as a secreted extracellular factor led us to determine whether recombinant human EPIM (rhEPIM) added to the culture media was capable of inducing a quiescent-like phenotype in activated rHSCs. Treatment of 7 day activated rHSCs with rhEPIM resulted in a dose-dependent decrease in SOX9, COLI and α-SMA proteins (Fig. 2A). 2 µg/ml rhEPIM reduced this pro-fibrotic protein profile by >60% from control levels (Fig. 2A). rhEPIM (2 µg/ml) also reduced a OPN levels by 65% (Fig. 2B). These data are remarkably similar to culturing activated HSCs on Matrigel [31]–[34], known to contain EPIM [35], rather than tissue culture plastic (Fig. 2C–D). These data concur with previous studies that have indicated activated HSCs cultured on soft substrates such as Matrigel can be ‘deactivated’ to a more quiescent-like state with greatly reduced α-SMA and COL1 expression [31]–[34].

### Epimorphin increases expression of proteases that cause collagen breakdown

**Figure 3:**
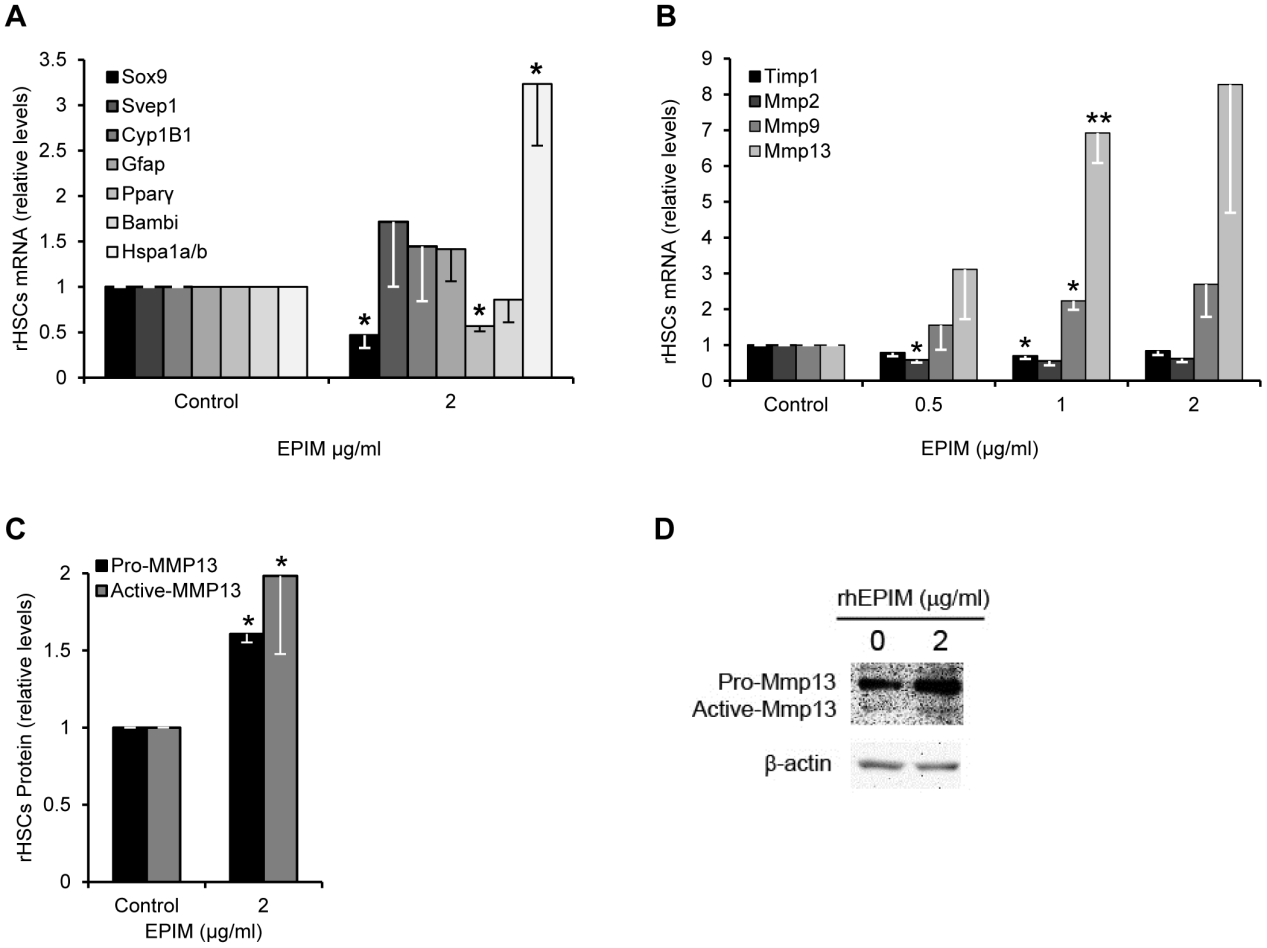
EPIM increases *Mmp13* expression in activated HSC. (A and B) Quantification of gene expression by qPCR in rhEPIM-treated activated rHSCs. *Hspa1a/b* expression was significantly increased (A), as were the proteases *Mmp9* and *Mmp13* (B) in response to rhEPIM. (C) Quantification of pro and active forms of MMP13 protein in rhEPIM-treated activated rHSCs. Both pro and active MMP13 protein levels were significantly increased. Example immunoblot shown in (D). *, p<0.05 and **, p<0.01.

To characterize rhEPIM-treated HSCs further we investigated the expression of genes previously associated with an inactive or deactivated HSC phenotype as indicative of the recovery phase following liver injury [36], [37]. We detected few changes in gene expression characteristic of this phenomenon (i.e. increased *Gfap*, *Pparγ* or *Bambi*; or decreased *Svep1* or *Cyp1B1*). EPIM did increase expression of pro-survival gene *Hspa1a/b* (Fig. 3A). However, there was no difference in total HSP70 protein, encompassing HSPA1a/b, or any difference in apoptosis as determined by the ratio of full length to cleaved Caspase 3 or proliferation determined by BrdU incorporation (Fig. S2A–C). In addition, activated HSCs treated with EPIM decreased *Pparγ* mRNA suggestive of a more myofibroblastic phenotype. However, we did not see any change in *Pparγ* gene expression at other concentrations of EPIM treatment (Fig. S2D) and the levels of *Pparγ* transcripts in activated HSCs were already very low. In line with this there was no change in gross cell morphology or lipid accumulation by oil red O staining in EPIM treated HSCs (data not shown).

Studies have suggested Epim facilitates the recovery phase following liver injury [22]. It has been associated with resolution of fibrosis in both kidney and liver by inducing protease expression to cause ECM degradation [22], [24]. In line with this, we detected altered MMP/TIMP expression in activated rHSCs treated with rhEPIM (Fig. 3B). Whereas expression of *Timp1* and *Mmp2* mRNA appeared to decrease following rhEPIM treatment, levels of *Mmp9* and *Mmp13* mRNA were increased (Fig. 3B). 1 µg/ml of rhEPIM significantly induced *Mmp9* and *Mmp13* by 2.7 and 7.2 fold respectively. Similarly, MMP13 protein was increased following rhEPIM treatment (pro-MMP13 by 1.6 fold and active-MMP13 by 2 fold; Fig. 3C & D). In rodents MMP13 is the primary interstitial collagenase and thought to be a key enzyme involved in ECM resolution [4]. During liver fibrosis, MMP13 is thought to be transiently increased during initial stages but becomes almost undetectable with progressive disease, coincident with an increase in TIMPs 1 & 2 [38]–[40]. In activated HSCs, both pro and active MMP13 were decreased (40% and 30% respectively; Fig. S3A). Conversely, similar to EPIM-treated activated HSCs, culturing cells on Matrigel increased pro and, to a lower extent, active forms of MMP13 (Fig. S3B).

### SOX9 limits levels of Epim and Mmp13 in activated HSCs

**Figure 4:**
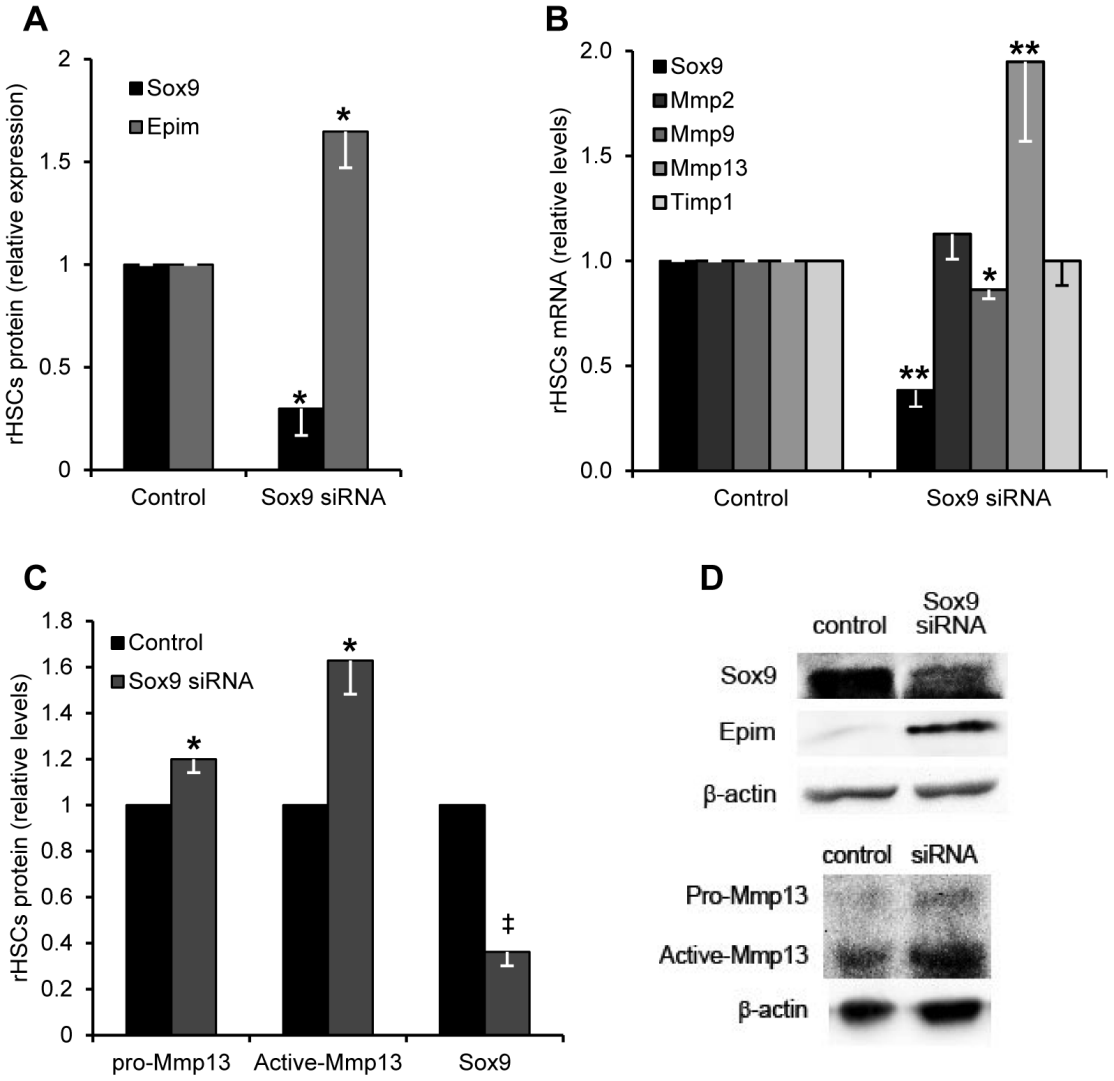
SOX9 knockdown increases EPIM and MMP13 expression in activated HSCs. Quantification of proteins (A and C) and genes (B) following siRNA abrogation of SOX9 in activated rHSCs standardized against scrambled siRNA control. Abrogation of SOX9 increases EPIM protein (A) and *Mmp13* mRNA and protein (B and C). (D) Example immunoblot shown for (A) and (C). Immunoblotting quantification was normalized to β-actin. *, p<0.05 and **, p<0.01.

**Figure 5:**
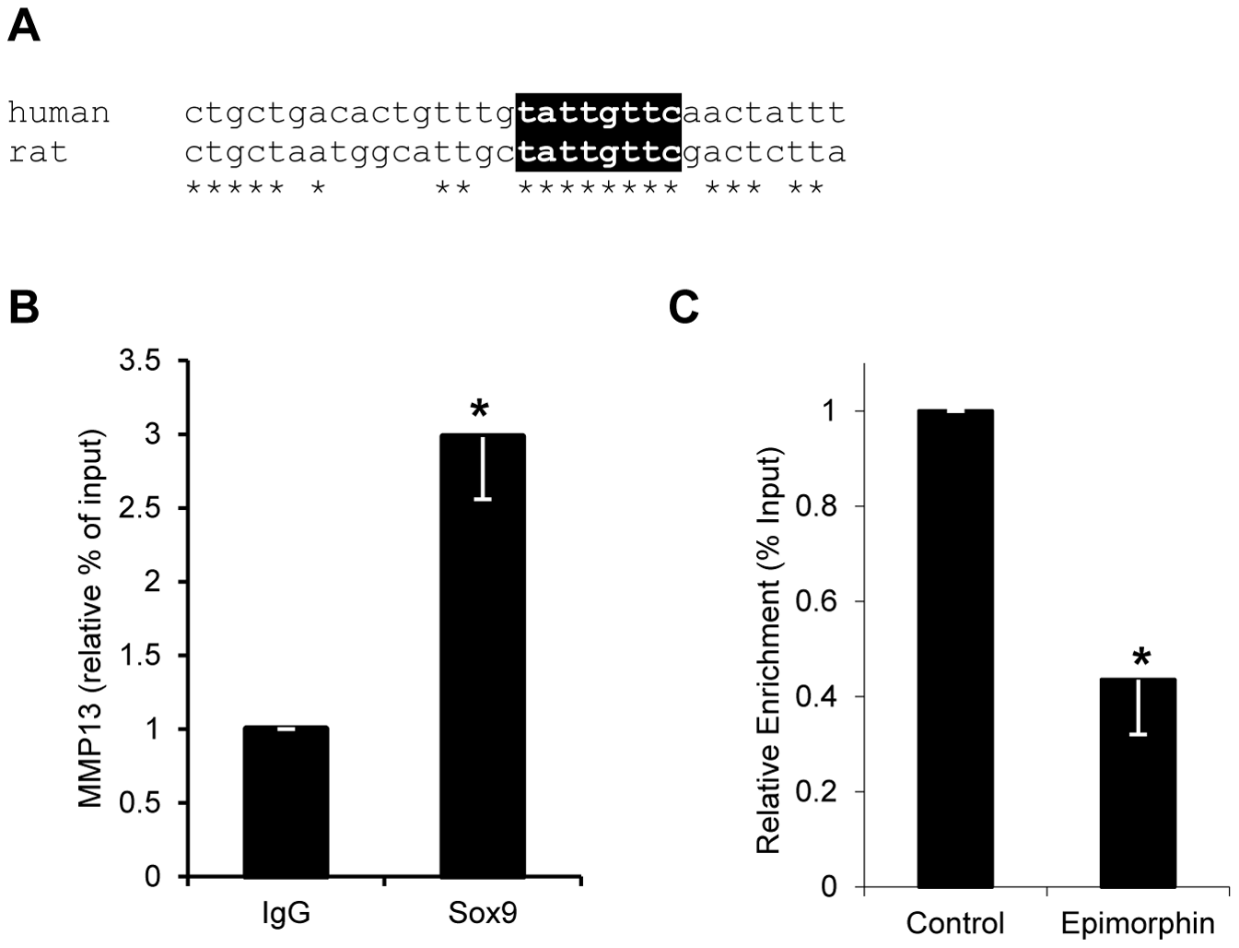
Sox9 directly binds to *Mmp13* in activated HSCs. (A) Alignment of a region within intron 7 of the *MMP13* gene with conserved SOX9-binding motif highlighted in black. Conserved nucleotides are indicated by asterisks (*). (B and C) ChIP-qPCR for SOX9-binding element in conserved region of *MMP13* in activated rHSCs. (B) Fold enrichment of *MMP13* over IgG negative control antibody (IgG) is shown for SOX9-bound region (SOX9). (C) Relative change in enrichment (% Input) of the SOX9-binding element in control versus rhEPIM treated activated rHSCs. *, p<0.05.

Given the reduction in EPIM as SOX9 becomes expressed in activated HSCs (Fig. 1A–B); we investigated whether EPIM was a downstream target of SOX9. *Sox9* silencing resulted in a 1.6 fold induction of EPIM protein (Fig. 4A & D). In silico analysis did not identify a SOX9 element within 10 kb of the human *EPIM* gene. However, although SOX9 siRNA treated activated HSCs demonstrated little change in the profibrotic enzymes *Mmp2*, *Mmp9* and *Timp1*, there was a significant >2 fold increase in *Mmp13* mRNA (Fig. 4B). Similarly, pro and active MMP13 protein was increased >1.2 and 1.5 fold respectively in response to *Sox9* abrogation (Fig. 4C and D). A conserved SOX9 binding motif was identified in intron 7 of *MMP13* (Fig. 5A) which bound SOX9 with a 3-fold enrichment following SOX9 ChIP (Fig. 5B). In contrast, treatment of HSCs with rhEPIM reduced the association of endogenous SOX9 with the *MMP13* gene by 56%.

## Discussion

**Figure 6:**
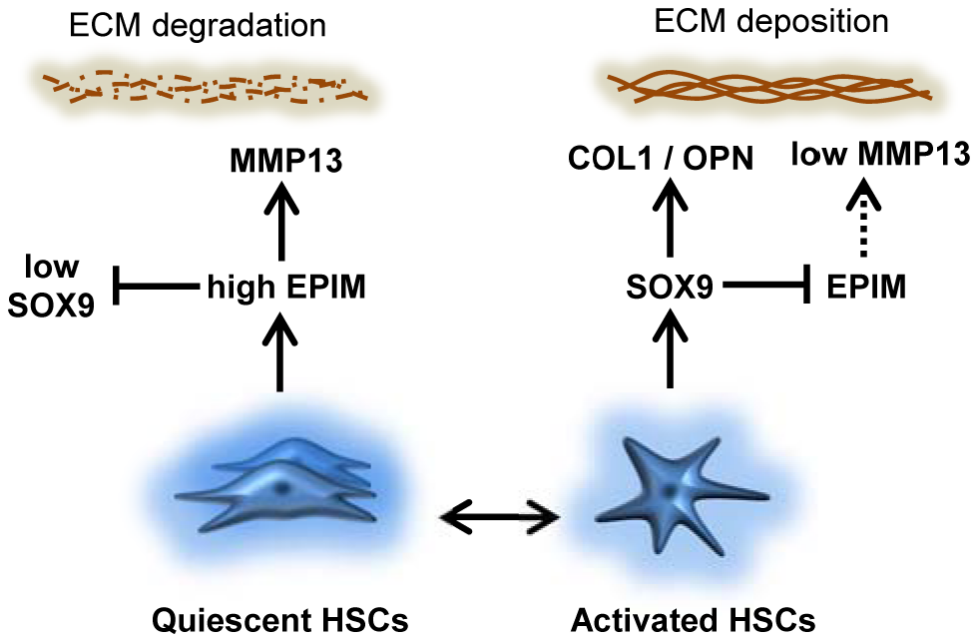
Schematic representation of the opposing roles of SOX9 and EPIM during liver fibrosis. The extracellular environment in the uninjured liver contains high levels of EPIM in quiescent HSCs and maintains a balance favoring ECM degradation through SOX9 inhibition and MMP13 expression. In contrast, as HSCs become activated following injury to the liver, EPIM is greatly reduced by the presence of SOX9 which favors ECM deposition rather than degradation. In addition to what is depicted via EPIM, high and low levels of SOX9 may directly repress and de-repress MMP13 expression respectively.

Liver fibrosis is a common step in the progression of the majority of chronic liver diseases. Despite this, there are no approved therapies to reduce or remove the characteristic scar associated with tissue damage. EPIM has been implicated in tissue repair mechanisms following injury to lung, kidney or liver [18], [22], [24], [41]. Previously, we have described how the transcription factor SOX9 regulates profibrotic proteins COL1 and OPN, in activated HSCs [6], [7]. In this study, we show that EPIM ameliorates the profibrotic phenotype of HSCs and, specifically, decreases SOX9 levels. Conversely, we demonstrate that the presence of SOX9 in activated HSCs limits the amount of EPIM production. Taken together these data support a model of inter-dependent states of high and low EPIM and SOX9 in determining whether ECM is deposited or degraded in liver fibrosis (Figure 6).

Our investigations were prompted by reports that EPIM was increased in the recovery phase following liver injury [22] when some HSCs are proposed to escape apoptosis and revert to a quiescent-like state [36], [37]. However, although our analysis of genes reported as characteristic of inactive or reverted HSCs [36], [37] only detected an increase in the pro-survival gene *Hspa1a/b*, we detected no change in HSP70 protein expression in rhEPIM-treated HSCs or any difference in apoptosis by Caspase 3 expression or proliferation by BrdU incorporation. Of interest, we also detected a decrease in the adipogenic gene *Pparγ*, associated with vitamin A uptake and lipid droplet formation in quiescent HSCs. Although, transcript levels of *Pparγ* in activated HSCs were already low potentially reflecting the detection limit for this study, the reduced availability of growth factors using serum free conditions may have also influenced *Pparγ* expression levels. However, in support of this we did not observed any change in gross cell morphology or lipid accumulation by oil red O staining. Moreover, our data showed that the more quiescent-like phenotype induced by EPIM in vitro is defined by reduced expression of the profibrotic genes *Sox9*, *α-Sma*, *Col1* and *Opn*; similar to our observations from culturing activated HSCs on Matrigel. EPIM also increased *Mmp13* expression. Thus, our data imply EPIM's major role in cells adopting a quiescent phenotype is in altering the balance between the production of collagen and a protease that digests it [21], [22], [24]. In line with this, we also detected more modest alterations in proteases favoring a less fibrotic phenotype with reduced *Timp1* and *Mmp2* and increased *Mmp9* expression in response to EPIM.

Similar to rhEPIM treated HSCs, elevated EPIM following SOX9 abrogation also increased *Mmp13* expression. Given that SOX9 was capable of directly binding to a conserved motif in the *MMP13* gene; potentially SOX9's effect on lowering MMP13 expression in activated HSCs could be either direct, indirect via Epim or both. Our data are concordant with chondrogenesis during development when SOX9 plays a key role in regulating cell proliferation and the expression of cartilage matrix genes [5]; in the later stages of chondrocyte maturation SOX9 is absent concomitant with an increase in MMP13 [42], [43]. In contrast to our data, retroviral transfer of SOX9 in chick fibroblasts increased EPIM expression in cells cultured as pellets [21]. However, no change in EPIM was detected in response to SOX9 transduction of cells cultured in monolayer. In our studies, we would hypothesize that culturing activated HSCs on Matrigel would induce re-expression of EPIM. Unfortunately we could not test this because Matrigel contained confounding amounts of EPIM.

Taken together, our data support a broad role for SOX9 in ECM deposition (e.g. increased COL1 and OPN) and inhibiting degradation, Epim-induced mediated by proteases. Given there are currently no approved effective antifibrotic drugs to treat liver fibrosis [2], [44], this study raises clinical possibilities. For instance, as a potential antifibrotic target, studies suggest enhanced expression of MMP13 attenuates liver fibrosis with reduced collagen levels [45], [46]. Attenuation of SOX9 might represent a more attractive target capable of both reducing scar formation by decreasing ECM deposition and increasing its degradation.

## Supporting Information

Figure S1 **Liver Fibrosis in CCl_4_ treated mice.** (A) Picro Sirius Red staining shows increased collagen deposition in liver sections from CCl_4_ treated mice (dark grey areas of scar), versus vehicle control (Oo – Olive Oil). (B) Immunoblot showing increased α-SMA protein in whole liver lysate from mice treated with CCl_4_ versus vehicle control (Oo). Loading control is β-actin.
(TIF)

Figure S2 **HSPA1a/b levels, apoptosis and proliferation are unchanged following rhEPIM treatment.** (A and B) Immunoblots showing two independent experiments of activated rHSCs treated with 2 µg/ml of rhEPIM. (A) HSPA1a/b was unchanged. β-actin control is shown for protein loading. (B) no alteration in full length or cleaved Caspase 3 was detected in response to rhEPIM. (C) activated rHSC proliferation was unchanged following EPIM treatment (2 µg/ml) indicated by cell numbers incorporating BrdU. Expressed as a percent of total cell numbers (n = 4). (D) Quantification of gene expression by qPCR in 0.5 µg/ml and 1 µg/ml rhEPIM-treated activated rHSCs (*, p<0.05).
(TIF)

Figure S3 **MMP13 expression is reduced in activated HSCs.** (A and B) Quantification of MMP13 in rHSCs. (A) Reduction in pro and active forms of MMP13 following activation of rHSCs. (B) Increased expression of both pro and active-MMP13 in activated rHSCs cultured on Matrigel for 14 days. Example immunoblots are shown in inset (A and B). *, p<0.05, ***, p<0.005.
(TIF)

Table S1 **qPCR primers.**
(DOC)

Table S2 **Antibodies and dilutions for immunoblotting.**
(DOC)

## References

[1] Cohen-Naftaly M, Friedman SL (2011) Current status of novel antifibrotic therapies in patients with chronic liver disease. Therap Adv Gastroenterol 4: 391–417. 10.1177/1756283X11413002 PMC3187682 22043231

[2] Friedman SL, Sheppard D, Duffield JS, Violette S (2013) Therapy for fibrotic diseases: nearing the starting line. Sci Transl Med 5: 167sr161. 10.1126/scitranslmed.3004700 23303606

[3] Friedman SL (2008) Mechanisms of hepatic fibrogenesis. Gastroenterology 134: 1655–1669. 18471545 10.1053/j.gastro.2008.03.003 PMC2888539

[4] Iredale JP (2007) Models of liver fibrosis: exploring the dynamic nature of inflammation and repair in a solid organ. J Clin Invest 117: 539–548. 17332881 10.1172/JCI30542 PMC1804370

[5] Pritchett J, Athwal V, Roberts N, Hanley NA, Hanley KP (2011) Understanding the role of SOX9 in acquired diseases: lessons from development. Trends Mol Med 17: 166–174. 21237710 10.1016/j.molmed.2010.12.001

[6] Piper Hanley K, Oakley F, Sugden S, Wilson DI, Mann DA, et al (2008) Ectopic SOX9 Mediates Extracellular Matrix Deposition Characteristic of Organ Fibrosis. Journal of Biological Chemistry 283: 14063–14071. 18296708 10.1074/jbc.M707390200

[7] Pritchett J, Harvey E, Athwal V, Berry A, Rowe C, et al (2012) Osteopontin is a novel downstream target of SOX9 with diagnostic implications for progression of liver fibrosis in humans. Hepatology 56: 1108–1116. 22488688 10.1002/hep.25758 PMC3638324

[8] Huang W, Zhu G, Huang M, Lou G, Liu Y, et al (2010) Plasma osteopontin concentration correlates with the severity of hepatic fibrosis and inflammation in HCV-infected subjects. Clin Chim Acta 411: 675–678. 20138033 10.1016/j.cca.2010.01.029

[9] Xie H, Song J, Du R, Liu K, Wang J, et al (2007) Prognostic significance of osteopontin in hepatitis B virus-related hepatocellular carcinoma. Dig Liver Dis 39: 167–172. 17161983 10.1016/j.dld.2006.10.015

[10] Zhao L, Li T, Wang Y, Pan Y, Ning H, et al (2008) Elevated plasma osteopontin level is predictive of cirrhosis in patients with hepatitis B infection. Int J Clin Pract 62: 1056–1062. 17537188 10.1111/j.1742-1241.2007.01368.x

[11] Syn WK, Choi SS, Liaskou E, Karaca GF, Agboola KM, et al (2011) Osteopontin is induced by hedgehog pathway activation and promotes fibrosis progression in nonalcoholic steatohepatitis. Hepatology 53: 106–115. 20967826 10.1002/hep.23998 PMC3025083

[12] Iredale JP (2001) Hepatic stellate cell behavior during resolution of liver injury. Semin Liver Dis 21: 427–436. 11586470 10.1055/s-2001-17557

[13] Hirai Y, Takebe K, Takashina M, Kobayashi S, Takeichi M (1992) Epimorphin: a mesenchymal protein essential for epithelial morphogenesis. Cell 69: 471–481. 1581962 10.1016/0092-8674(92)90448-l

[14] Radisky DC, Hirai Y, Bissell MJ (2003) Delivering the message: epimorphin and mammary epithelial morphogenesis. Trends Cell Biol 13: 426–434. 12888295 10.1016/s0962-8924(03)00146-6 PMC2933193

[15] Jia Y, Yao H, Zhou J, Chen L, Zeng Q, et al (2011) Role of epimorphin in bile duct formation of rat liver epithelial stem-like cells: involvement of small G protein RhoA and C/EBPbeta. J Cell Physiol 10.1002/jcp.22625 21935930

[16] Zhou J, Zhao L, Qin L, Wang J, Jia Y, et al (2011) Epimorphin regulates bile duct formation via effects on mitosis orientation in rat liver epithelial stem-like cells. PLoS One 5: e9732. 10.1371/journal.pone.0009732 PMC2840022 20305811

[17] Radisky DC, Stallings-Mann M, Hirai Y, Bissell MJ (2009) Single proteins might have dual but related functions in intracellular and extracellular microenvironments. Nat Rev Mol Cell Biol 10: 228–234. 19190671 10.1038/nrm2633 PMC2746016

[18] Segawa D, Miura K, Goto T, Ohshima S, Mikami K, et al (2005) Distribution and isoforms of epimorphin in carbon tetrachloride-induced acute liver injury in mice. J Gastroenterol Hepatol 20: 1769–1780. 16246199 10.1111/j.1440-1746.2005.03944.x

[19] Hirose M, Watanabe S, Oide H, Kitamura T, Miyazaki A, et al (1996) A new function of Ito cells in liver morphogenesis: evidence using a novel morphogenic protein, epimorphin, in vitro. Biochem Biophys Res Commun 225: 155–160. 8769110 10.1006/bbrc.1996.1146

[20] Zhou J, Zhao L, Qin L, Wang J, Jia Y, et al (2010) Epimorphin regulates bile duct formation via effects on mitosis orientation in rat liver epithelial stem-like cells. PLoS One 5: e9732. 20305811 10.1371/journal.pone.0009732 PMC2840022

[21] Yoshino R, Miura K, Segawa D, Hirai Y, Goto T, et al (2006) Epimorphin expression and stellate cell status in mouse liver injury. Hepatol Res 34: 238–249. 16480920 10.1016/j.hepres.2005.12.011

[22] Miura K, Yoshino R, Hirai Y, Goto T, Ohshima S, et al (2007) Epimorphin, a morphogenic protein, induces proteases in rodent hepatocytes through NF-kappaB. J Hepatol 47: 834–843. 17935821 10.1016/j.jhep.2007.07.024

[23] Watanabe S, Hirose M, Wang XE, Ikejima K, Oide H, et al (1998) A novel hepatic stellate (Ito) cell-derived protein, epimorphin, plays a key role in the late stages of liver regeneration. Biochem Biophys Res Commun 250: 486–490. 9753658 10.1006/bbrc.1998.9339

[24] Yamada M, Oda T, Higashi K, Kushiyama T, Yamakami K, et al (2010) Involvement of epimorphin in the repair of experimental renal fibrosis in mice. Lab Invest 90: 867–880. 20195239 10.1038/labinvest.2010.50

[25] Jia YL, Shi L, Zhou JN, Fu CJ, Chen L, et al (2011) Epimorphin promotes human hepatocellular carcinoma invasion and metastasis through activation of focal adhesion kinase/extracellular signal-regulated kinase/matrix metalloproteinase-9 axis. Hepatology 54: 1808–1818. 22045676 10.1002/hep.24562

[26] Smart DE, Green K, Oakley F, Weitzman JB, Yaniv M, et al (2006) JunD is a profibrogenic transcription factor regulated by Jun N-terminal kinase-independent phosphorylation. Hepatology 44: 1432–1440. 17133482 10.1002/hep.21436

[27] Rowe C, Gerrard DT, Jenkins R, Berry A, Durkin K, et al (2013) Proteome-wide analyses of human hepatocytes during differentiation and dedifferentiation. Hepatology 58: 799–809. 23526496 10.1002/hep.26414 PMC3842115

[28] Oakley F, Meso M, Iredale JP, Green K, Marek CJ, et al (2005) Inhibition of inhibitor of kappaB kinases stimulates hepatic stellate cell apoptosis and accelerated recovery from rat liver fibrosis. Gastroenterology 128: 108–120. 15633128 10.1053/j.gastro.2004.10.003

[29] Piper Hanley K, Hearn T, Berry A, Carvell MJ, Patch AM, et al (2010) In vitro expression of NGN3 identifies RAB3B as the predominant Ras-associated GTP-binding protein 3 family member in human islets. J Endocrinol 207: 151–161. 20807725 10.1677/JOE-10-0120 PMC2951179

[30] Donaldson IJ, Amin S, Hensman JJ, Kutejova E, Rattray M, et al (2012) Genome-wide occupancy links Hoxa2 to Wnt-beta-catenin signaling in mouse embryonic development. Nucleic Acids Res 40: 3990–4001. 22223247 10.1093/nar/gkr1240 PMC3351182

[31] Friedman SL, Roll FJ, Boyles J, Arenson DM, Bissell DM (1989) Maintenance of differentiated phenotype of cultured rat hepatic lipocytes by basement membrane matrix. J Biol Chem 264: 10756–10762. 2732246

[32] Gaca MD, Zhou X, Issa R, Kiriella K, Iredale JP, et al (2003) Basement membrane-like matrix inhibits proliferation and collagen synthesis by activated rat hepatic stellate cells: evidence for matrix-dependent deactivation of stellate cells. Matrix Biol 22: 229–239. 12853033 10.1016/s0945-053x(03)00017-9

[33] Shimada H, Rajagopalan LE (2012) Employment of gene expression profiling to identify transcriptional regulators of hepatic stellate cells. Fibrogenesis Tissue Repair 5 Suppl 1: S12. 23259668 10.1186/1755-1536-5-S1-S12 PMC3368757

[34] Sohara N, Znoyko I, Levy MT, Trojanowska M, Reuben A (2002) Reversal of activation of human myofibroblast-like cells by culture on a basement membrane-like substrate. J Hepatol 37: 214–221. 12127426 10.1016/s0168-8278(02)00103-4

[35] Hirai Y (1993) Molecular cloning of human epimorphin: identification of isoforms and their unique properties. Biochem Biophys Res Commun 191: 1332–1337. 8466509 10.1006/bbrc.1993.1363

[36] Kisseleva T, Cong M, Paik Y, Scholten D, Jiang C, et al (2012) Myofibroblasts revert to an inactive phenotype during regression of liver fibrosis. Proc Natl Acad Sci U S A 109: 9448–9453. 22566629 10.1073/pnas.1201840109 PMC3386114

[37] Troeger JS, Mederacke I, Gwak GY, Dapito DH, Mu X, et al (2012) Deactivation of hepatic stellate cells during liver fibrosis resolution in mice. Gastroenterology 143: 1073–1083 e1022. 22750464 10.1053/j.gastro.2012.06.036 PMC3848328

[38] Iredale JP, Murphy G, Hembry RM, Friedman SL, Arthur MJ (1992) Human hepatic lipocytes synthesize tissue inhibitor of metalloproteinases-1. Implications for regulation of matrix degradation in liver. J Clin Invest 90: 282–287. 1634616 10.1172/JCI115850 PMC443094

[39] Kossakowska AE, Edwards DR, Lee SS, Urbanski LS, Stabbler AL, et al (1998) Altered balance between matrix metalloproteinases and their inhibitors in experimental biliary fibrosis. Am J Pathol 153: 1895–1902. 9846979 10.1016/S0002-9440(10)65703-3 PMC1866318

[40] Watanabe T, Niioka M, Hozawa S, Kameyama K, Hayashi T, et al (2000) Gene expression of interstitial collagenase in both progressive and recovery phase of rat liver fibrosis induced by carbon tetrachloride. J Hepatol 33: 224–235. 10952240 10.1016/s0168-8278(00)80363-3

[41] Terasaki Y, Fukuda Y, Ishizaki M, Yamanaka N (2000) Increased expression of epimorphin in bleomycin-induced pulmonary fibrosis in mice. Am J Respir Cell Mol Biol 23: 168–174. 10919982 10.1165/ajrcmb.23.2.3973

[42] Hattori T, Muller C, Gebhard S, Bauer E, Pausch F, et al (2010) SOX9 is a major negative regulator of cartilage vascularization, bone marrow formation and endochondral ossification. Development 137: 901–911. 20179096 10.1242/dev.045203

[43] Nishimura R, Wakabayashi M, Hata K, Matsubara T, Honma S, et al (2012) Osterix regulates calcification and degradation of chondrogenic matrices through matrix metalloproteinase 13 (MMP13) expression in association with transcription factor Runx2 during endochondral ossification. J Biol Chem 287: 33179–33190. 22869368 10.1074/jbc.M111.337063 PMC3460424

[44] Friedman SL (2003) Liver fibrosis – from bench to bedside. J Hepatol 38 Suppl 1: S38–53. 12591185 10.1016/s0168-8278(02)00429-4

[45] Endo H, Niioka M, Sugioka Y, Itoh J, Kameyama K, et al (2011) Matrix metalloproteinase-13 promotes recovery from experimental liver cirrhosis in rats. Pathobiology 78: 239–252. 21849805 10.1159/000328841

[46] Kim EJ, Cho HJ, Park D, Kim JY, Kim YB, et al (2011) Antifibrotic effect of MMP13-encoding plasmid DNA delivered using polyethylenimine shielded with hyaluronic acid. Mol Ther 19: 355–361. 21139571 10.1038/mt.2010.262 PMC3034855

