# Supplementary figures and images for: Epimorphin Alters the Inhibitory Effects of SOX9 on Mmp13 in Activated Hepatic Stellate Cells
Source: PLoS One. 2014 Jun 27;9(6):e100091. doi: 10.1371/journal.pone.0100091 (PMC4074045; doi:10.1371/journal.pone.0100091)

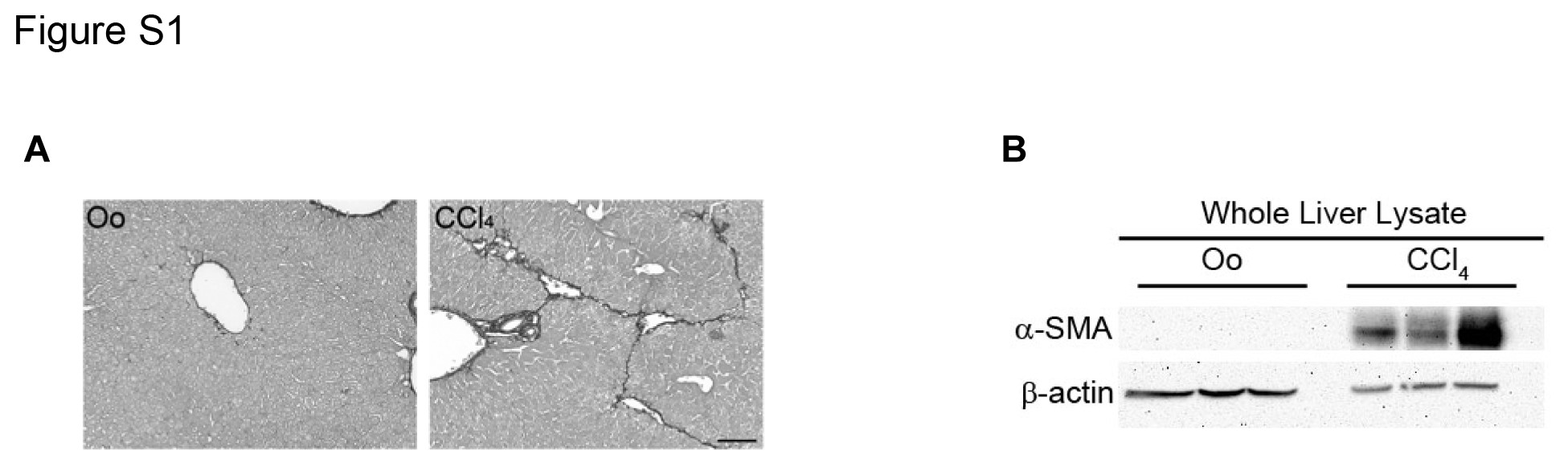

Supplement: Figure S1 — Liver Fibrosis in CCl4 treated mice. (A) Picro Sirius Red staining shows increased collagen deposition in liver sections from CCl4 treated mice (dark grey areas of scar), versus vehicle control (Oo – Olive Oil). (B) Immunoblot showing increased α-SMA protein in whole liver lysate from mice treated with CCl4 versus vehicle control (Oo). Loading control is β-actin. (TIF) [file pone.0100091.s001.tif]

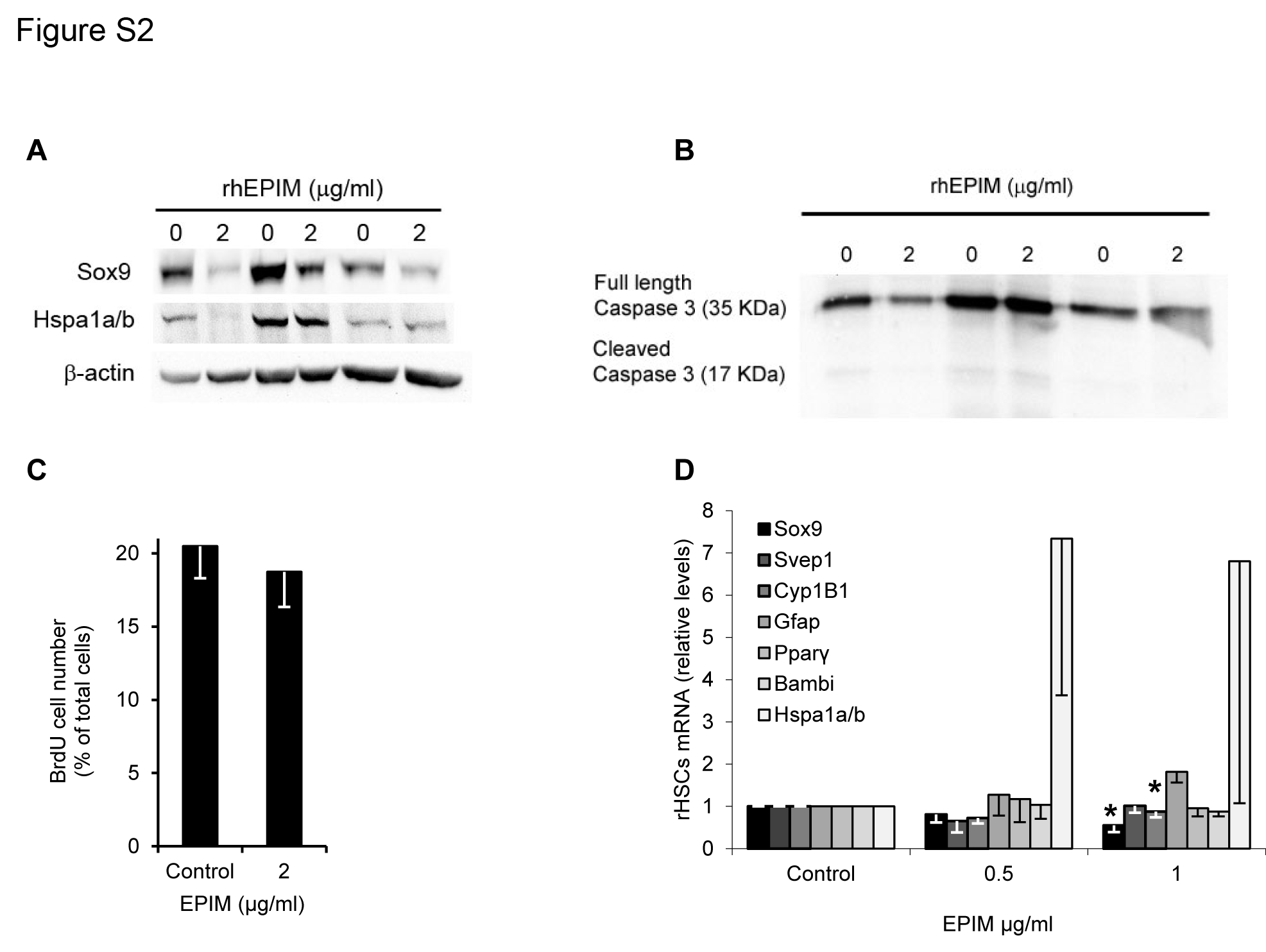

Supplement: Figure S2 — HSPA1a/b levels, apoptosis and proliferation are unchanged following rhEPIM treatment. (A and B) Immunoblots showing two independent experiments of activated rHSCs treated with 2 µg/ml of rhEPIM. (A) HSPA1a/b was unchanged. β-actin control is shown for protein loading. (B) no alteration in full length or cleaved Caspase 3 was detected in response to rhEPIM. (C) activated rHSC proliferation was unchanged following EPIM treatment (2 µg/ml) indicated by cell numbers incorporating BrdU. Expressed as a percent of total cell numbers (n = 4). (D) Quantification of gene expression by qPCR in 0.5 µg/ml and 1 µg/ml rhEPIM-treated activated rHSCs (*, p<0.05). (TIF) [file pone.0100091.s002.tif]

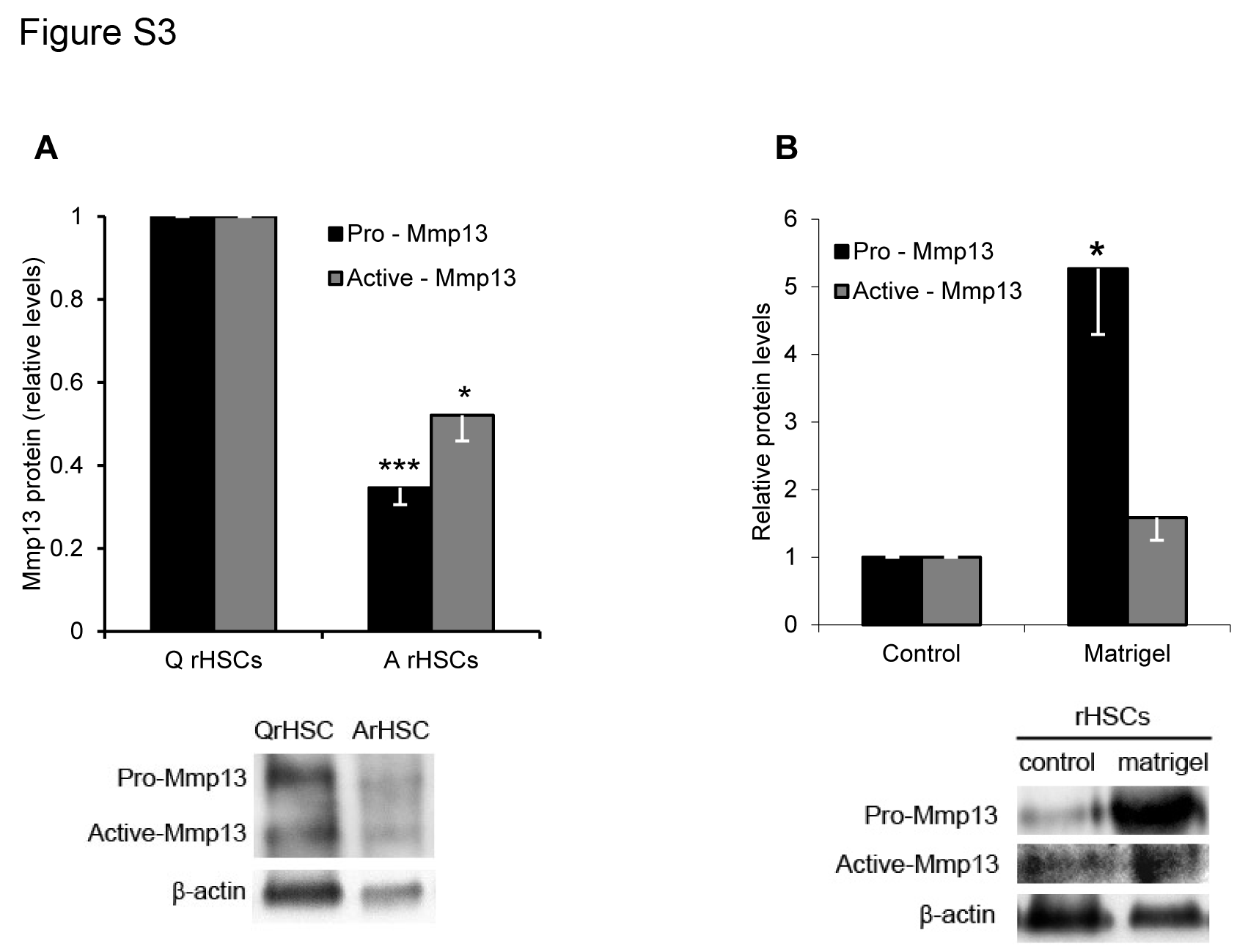

Supplement: Figure S3 — MMP13 expression is reduced in activated HSCs. (A and B) Quantification of MMP13 in rHSCs. (A) Reduction in pro and active forms of MMP13 following activation of rHSCs. (B) Increased expression of both pro and active-MMP13 in activated rHSCs cultured on Matrigel for 14 days. Example immunoblots are shown in inset (A and B). *, p<0.05, ***, p<0.005. (TIF) [file pone.0100091.s003.tif]
