# Supplementary material for: Epimorphin Alters the Inhibitory Effects of SOX9 on Mmp13 in Activated Hepatic Stellate Cells
Source: PLoS One. 2014 Jun 27;9(6):e100091. doi: 10.1371/journal.pone.0100091 (PMC4074045; doi:10.1371/journal.pone.0100091)
Supplement: Table S1 — qPCR primers. (DOC) [file pone.0100091.s004.doc]

Table S1. qPCR primers.

| Gene | Species | Forward | Reverse |
| --- | --- | --- | --- |
| *Sox9* | Rat | gcaagtagccctggtttcgttctc | gggtggccagtgctcagttgc |
| *GusB* | Rat | ctctggtggccttacctgat | aggtgttgtcatcgtcacctc |
| *ActinB* | Rat | cccgcgagtacaaccttct | cgtcatccatggcgaact |
| *Timp1* | Rat | ttccggttcgcctacacccca | tccttaaacggcccgcgatga |
| *Mmp2* | Rat | tttgctcgggccttaaaagtat | ccatcaaacgggtatccatctc |
| *Mmp9* | Rat | actcgagccgacgtcactgt | ggccctcgccggtacaggta |
| *Mmp13* | Rat | ctggaccaaaccttggcgggg | agttgtagcctttggagctgcttg |
| *Bambi* | Rat | tgtgctgctcaccaaaggcga | agcaggcactaagctcggact |
| *Gfap* | Rat | ccttgcgcggcacgaacgag | ccgagcgagtgcctcctggt |
| *Ppar γ* | Rat | tctcagtggagaccgcccagg | gggaggactccgggtggttcag |
| *Svep1* | Rat | tcccctgtttgaatggtggg | cttccgtcagacgtgactgt |
| *Cyp1b1* | Rat | gtatgcttcggctgtcggta | gaactcctcgttgtggctga |
| *Hspa1a/b* | Rat | tgtccctcaagagcccaaccc | ttggctctccacacaggaaccc |

### 
