# Supplementary material for: Epimorphin Alters the Inhibitory Effects of SOX9 on Mmp13 in Activated Hepatic Stellate Cells
Source: PLoS One. 2014 Jun 27;9(6):e100091. doi: 10.1371/journal.pone.0100091 (PMC4074045; doi:10.1371/journal.pone.0100091)
Supplement: Table S2 — Antibodies and dilutions for immunoblotting. (DOC) [file pone.0100091.s005.doc]

Table S2. Antibodies and dilutions for immunoblotting.

| **Antibody** | **Raised In** | **Company** | **Dilution** |
| --- | --- | --- | --- |
| SOX9 | Rabbit | Millipore | 1:5000 |
| OPN | Goat | Abcam | 1:750 |
| α-SMA | Mouse | Leica/Novocastra | 1:100 |
| β-actin | Mouse | Sigma | 1:100 000 |
| Col I | Rabbit | Gift from Dr Larry Fisher, NIH,  Bethesda, MD | 1:2000 |
| EPIM | Goat | R & D | 1:1000 |
| Hsp70 | Rabbit | Cell Signalling | 1:1000 |
| Caspase 3 | Rabbit | Cell Signalling | 1:1000 |
| MMP13 | Mouse | Millipore | 1:500 |
| BrdU | Sheep | Abcam (ab1893) | 1:1000 |
